# Does the implementation of an electronic prescribing system create unintended medication errors? A study of the sociotechnical context through the analysis of reported medication incidents

**DOI:** 10.1186/1472-6947-11-29

**Published:** 2011-05-12

**Authors:** Sabi Redwood, Anna Rajakumar, James Hodson, Jamie J Coleman

**Affiliations:** 1 University of Birmingham, School of Health and Population Sciences, Birmingham, Edgbaston Campus, B15 2TH, UK; 2 University Hospitals Birmingham NHS Foundation Trust, Edgbaston, Mindelsohn Way, Birmingham, B15 2PR, UK

## Abstract

**Background:**

Even though electronic prescribing systems are widely advocated as one of the most effective means of improving patient safety, they may also introduce new risks that are not immediately obvious. Through the study of specific incidents related to the processes involved in the administration of medication, we sought to find out if the prescribing system had unintended consequences in creating new errors. The focus of this study was a large acute hospital in the Midlands in the United Kingdom, which implemented a Prescribing, Information and Communication System (PICS).

**Methods:**

This exploratory study was based on a survey of routinely collected medication incidents over five months. Data were independently reviewed by two of the investigators with a clinical pharmacology and nursing background respectively, and grouped into broad types: sociotechnical incidents (related to human interactions with the system) and non-sociotechnical incidents. Sociotechnical incidents were distinguished from the others because they occurred at the point where the system and the professional intersected and would not have occurred in the absence of the system. The day of the week and time of day that an incident occurred were tested using univariable and multivariable analyses. We acknowledge the limitations of conducting analyses of data extracted from incident reports as it is widely recognised that most medication errors are not reported and may contain inaccurate data. Interpretation of results must therefore be tentative.

**Results:**

Out of a total of 485 incidents, a modest 15% (n = 73) were distinguished as sociotechnical issues and thus may be unique to hospitals that have such systems in place. These incidents were further analysed and subdivided into categories in order to identify aspects of the context which gave rise to adverse situations and possible risks to patient safety. The analysis of sociotechnical incidents by time of day and day of week indicated a trend for increased proportions of these types of incidents occurring on Sundays.

**Conclusion:**

Introducing an electronic prescribing system has the potential to give rise to new types of risks to patient safety. Being aware of these types of errors is important to the clinical and technical implementers of such systems in order to, where possible, design out unintended problems, highlight training requirements, and revise clinical practice protocols.

## Background

Patient safety is defined as the avoidance, prevention, and amelioration of adverse outcomes or injuries stemming from the process of health care [1]. Preventing medical errors is one of the most important aspects of providing safe and high quality care within health care systems.

Leape [2] defines error as an unintended act of either commission or omission that did not achieve its intended outcome. Significant research has been undertaken over the past few decades in studying issues around errors and adverse events. The delivery of medication to patients is the end result of a complex process comprising many steps, each vulnerable to error. Therefore in the acute hospital setting, preventing medical, and in particular medication errors, through the use of Information and Communication Technologies (ICT) is widely advocated as one of the most effective means of achieving such improvements [3,4]. Anticipated benefits include, for example, the reduction of legibility errors thus making prescribing safer, the increase in efficiency through eliminating lost notes or charts, improvements in communication between care providers and the increase in capacity at a time of workforce and financial constraint [4].

There is growing evidence from countries such as the United States where the use of health care ICT has a longer history, that even though electronic prescribing systems with inbuilt clinical decision support may contain features that protect against error and thus enhance patient safety [5], they may also introduce new risks of their own that are not always immediately obvious [6]. Some of the issues that have been identified are cognitive overload; loss of overview of the clinical situation; errors in data entry and retrieval; excessive reliance on electronically held data; disruptions of established workflow patterns and the tendency to infer that data entry equates to communication within and among health care teams [7,8]. These new risks are the unintended and unanticipated consequences associated with the introduction of the electronic prescribing system. There is a distinction to be drawn between the unintended and the unanticipated consequences in that "unintended" implies lack of purposeful action or causation, while "unanticipated" means an inability to forecast what eventually occurred [9]. Unintended errors can be exemplified by situations such as picking a wrong option from a drop down list or typing 100 instead of 10, which we consider to be akin to a slip which is a failure of attention leading to inadvertent actions. Lapses are failures of memory and often occur as a result of distractions or interruptions, whereas mistakes are caused by inappropriate clinical reasoning and failures in planning or problem solving [10]. In purely technological terms, an unanticipated consequence can occur when there are incorrect rules within a computer system or some other technical failure. Furthermore, unanticipated errors can occur if a user deliberately deviates from the standard procedures, recommendations or guidelines thus committing a technical violation [11] often referred to as a 'work-around' when there is a glitch in the way information systems are used relative to initial intentions [12,13]. While previous studies, predominantly North American, have produced some promising insights, given the fundamental differences in health care delivery, it is likely that they are not easily transferable into the UK context. Understanding the contribution of health care ICT to improving patient safety as well as its unanticipated and unintended consequences in the local and UK context is vital in order to facilitate the design of interventions at individual, team and organisational level to mitigate these potential new threats and thus further improve patient safety.

According to Shekelle and Goldzweig [6], computerised systems should be considered as a complex intervention with four key components: technical, human, project management, and organisational and cultural change all of which must be systematically studied. However, as Greenhalgh et al. [14] conclude in their extensive review of the literature of electronic patient records, research has only scratched the surface of what the introduction of clinical decision support systems means, at the level of fine-grained detail, for a health care organisation and the staff and patients who practise and interact in that setting. We propose an approach which acknowledges the mutual influence between technology and its social and organisational context [15,16] in which research focuses on the sociotechnical aspects (issues involving the interplay of organisational, individual, social and technical components) of patient safety and risk management in hospitals. New technology is not simply integrated into current practice and ways of working, but has a profound impact on organisational arrangements, professional work, and medical practice. This social view of technology recognises that individual systems are anchored to a variety of other practices as well as broader organisational conditions [17] which we investigated with respect to this locally developed system.

The purpose of this study was to explore the types of possible unintended and unanticipated consequences as well as the nature of their effects within the sociotechnical and organisational context at an acute hospital. To date, few hospitals in the UK have implemented a comprehensive IT system with the aim of reducing error and enhancing patient safety, and there is as yet little knowledge on the implications of such a highly computerised environment for working practices and care processes. In order to find out if the electronic prescribing system had unintended consequences in creating new errors, we accessed the existing risk management system to collect data in the form of reported incidents which were related to the medication process (i.e. prescribing, preparation, supply and administration of medicines). From a sociotechnical perspective, capturing this subjective contextual data was useful insofar as we sought to identify the dynamics between technology and the social, professional, and cultural environment in which it was used which in turn can be useful in developing preventative strategies [18]. However, many incidents may not be reported or may not be reported in enough detail regarding contributory factors and preceding events [19,20], or may contain inaccurate information.

Some recent UK based studies [21-23] have investigated medication errors related specifically to electronic prescribing systems by retrospectively reviewing electronic records for a random selection of patients, or by retrospectively analysing all medication orders generated in a specified period of time. In contrast, we worked with the hospital's risk management team, collecting all medication related clinical incidents, irrespective of their association with the electronic prescribing system, reported by hospital staff. Our objectives were to describe the range of medication incidents reported by hospital staff, identify the proportion of incidents that relate to sociotechnical factors and explore the nature and characteristics of reported sociotechnical incidents.

Based on the fact that clinical incident reports are not by their nature neutral descriptions when reported by individual members of health care staff, one of the foci of our analysis was the manner in which sociotechnical problems were described and presented in the incident reports, and what views and perceptions about the system they revealed. The study also allowed us to gain an insight into hospital staff's views of the system and its perceived role in mediating or preventing medication related errors. We did not seek to evaluate the technical performance of the system or investigate the validity of concerns expressed by clinical staff related to perceived technical problems.

## Methods

### Setting

The focus of this study was an acute hospital in the West Midlands in the United Kingdom (UK) which has fully implemented a comprehensive Prescribing, Information and Communication System (PICS) and provided a unique opportunity to investigate the effects of ICT on patient safety. The PICS system had been developed locally in collaboration between technical and clinical staff as an electronic prescribing, clinical decision support and alerting system that supports local working practices. As such, many of the difficulties associated with commercially-developed electronic prescribing systems [6], such as problems of fit with specific workflows or failure to meet the expectations of both clinical and managerial staff, had been largely avoided. Decision support was built into the rules-based system which included drug-drug interactions, drug-disease contraindications, dose range checking, drug-laboratory warnings, pregnancy / breast feeding / liver and renal warnings, and some structured orders relevant to local protocols (e.g. antibiotic prescribing). The system had been incrementally implemented across the organisation over a number of years and was well embedded into clinical practice across the organisation. The electronic prescribing system was used throughout all inpatient beds and across all specialties except for theatres, Accident and Emergency attendances and for the Day case / Ambulatory care unit, where the expected stay is less than 24 hours.

### Data collection

This study was based on a survey of routinely collected medication incident reports completed by members of staff through the hospital's clinical risk management system. At the time of data collection, the hospital had introduced software that enabled incident reports to be submitted online from wards and departments. Roll-out of the system across the hospital was not complete until month 3 of data collection. This meant that some of the early incident reports were completed on paper-based versions of the same reporting template and sent via the internal post to the risk management team who manually entered these data into the new system. Investigators did not detect any systematic differences in the data collected from the online or the paper-based systems. All hospital staff received guidance on incident reporting through policy and procedural documents requiring them to report all incidents, including near misses. An incident was defined as an unplanned or unexpected event that may or may not lead to injury, damage or loss to an individual or to the organisation. A near miss was defined as an event that had the potential to lead to injury damage or loss, but was prevented. The study hospital promoted a culture of openness to the reporting of incidents and near misses, and a report by the National Patient Safety Agency showed that it compared favourably with other organisations in a national cluster group in relation to medication incident reporting, based on an examination of patient safety incident reporting across the country and a comparison with similar institutions [24]. We only collected data from incidents that were medication related. The initial time period of 3 months was extended to 5 months as the actual rate of reported incidents was lower than originally estimated and we required a larger sample of incidents to conduct a statistical analysis. Data were extracted and collated by one of the investigators on a weekly basis in collaboration with the hospital's risk management department. The data had not been pre-coded. The following information relating to each incident was recorded by the researcher: hospital site; clinical area; time and date the incident took place; incident identification number; and the narrative description of the incident as given by the member of staff making the report. In all cases, the original words of the reporter were noted.

### Data analysis

The data were entered into a spreadsheet. Each incident was reviewed independently by two of the investigators (one of the investigators is a clinical pharmacologist, the other a registered nurse) in order to identify the incident's salient features and allocate it to a broad type (see below) and to a descriptive category (for example, related to the supply of medication, the prescribing process, or the administration of medicines). Reviewer disagreements were rare; when they occurred incidents were discussed and disagreements resolved. No further attempts were made to identify individual or technical circumstances relating to individual errors. Following the clinical reviews, the incidents were grouped into broad types: sociotechnical incidents and non-sociotechnical incidents. Sociotechnical incidents were distinguished from the other incidents because they were associated with the human interface and sociotechnical context of the electronic prescribing system. These incidents occurred at the point where the electronic prescribing system and the health care professional as the user of the system intersected and would not have occurred in the absence of the system. The two broad types of incidents were then broken down into more specific categories in order to identify the frequency with which particular problems occurred, using an iterative and inductive process for the categorisation in order to account for all incidents. Finally, each sociotechnical incident was examined in more detail in order to study how the incident was presented and described.

We further analysed the sociotechnical incidents by examining the overall characteristics of this subset in relation to the time of day and the day of the week they took place in order to uncover any underlying trends in incident occurrence. Initially, the effect of the time of day and day of the week were tested by univariable analysis, before both factors were considered together in a multivariable analysis. Chi-squared and Fisher's Exact tests were used to analyse the differences in the proportions of total incidents which were sociotechnical by day of the week, and by the reported time (08.00 to 18.00 were considered as normal working hours on both weekdays and weekends). A multivariable analysis was then conducted using a backwards stepwise logistic regression to analyse these factors simultaneously and to consider potential interactions.

### Approvals and permissions

All patient and staff names were removed at the data extraction stage in order to maintain anonymity. The survey was approved by the clinical audit department at the hospital.

## Results

Over a five month period a total of 485 medication incident reports were collected. The average number of incidents reported per day was 3.2 equating to an average of 23 per week. Two incidents had fatal outcomes (occurrence of these incidents was not directly attributed to cause of death) and a further five were classed as 'near misses' (i.e. incidents that had the potential to cause harm, but were prevented). This term was either used by the reporters themselves, or the reporter described an incident fitting the definition.

### Description of incidents

Out of a total of 485 reported incidents, 85% (n = 412) were unrelated to the presence of the electronic prescribing system within the medication use process (i.e. part of the whole general process from prescribing to administration or supply of medicines) and thus were not connected to the introduction of ICT in the study hospital. These included incidents that were associated with ward/ department drug stock or the supply of medicines to wards/ departments (n = 158, 32.6%), the administration of medicines (n = 123, 25.4%) and a group of a wide range of infrequently occurring incidents (27%, n = 131) which were related to controlled drugs/ narcotics, staff communication, patient identification, omissions, adverse reactions and infusion equipment. Fifteen per cent (n = 73) were distinguished as sociotechnical issues, representing the third highest category of all reported incidents, and thus may be unique to hospitals that have such ICT systems in place. These sociotechnical incidents were further analysed and subdivided into categories in order to identify aspects of the context which gave rise to adverse situations and which present possible risks to patient safety.

• ***Incidents related to missing electronic signatures on administration***

This category, which accounted for 49% (n = 36) of the sociotechnical incidents, describes incidents in which electronic signatures were not recorded against prescriptions in the administration section of the patient's medication records. On some occasions, the nurse who was called to account for the lack of electronic signature stated that she or he had placed the electronic signature, but that it had not registered on the system. These incidents were usually identified by staff coming on duty after the omission of signature took place. They responded in the following ways: they checked with the member of staff responsible for drug administration whether the drug had been given. However, they were not able to verify administration or omission on all occasions. In some incidents, staff had been told that the nurse responsible for the administration believed that an electronic signature had indeed been placed after the medicine had been given to the patient, but had not registered on the system and they therefore described it as a technical problem. In other cases, the lack of electronic signature was interpreted as signifying that the medicine had not been given to the patient and the medication was administered as if it had previously been omitted. With regard to the latter, on some occasions the patient or a relative spotted the potential for a double administration and informed the nurse about to administer the medication; in other cases a double administration occurred and was discovered in retrospect. Although this type of incident could be defined as a technical lapse, which would make it eligible for the following category of incidents which are related to technical slips and lapses, we have categorised it separately because of the relatively large number of this type of incident reported.

• ***Incidents related to technical slips or lapses during the prescribing or administration process***

Twenty three incidents (31%) were of this type, in which an error occurred within the prescribing or administration part of the process; in other words, they happened as a direct result of the system being used in the clinical environment, and would not have occurred in the same way, using traditional paper-based prescribing and administration systems. They are not a reflection on the system's technical quality, but indicate that it introduces the potential for new technical slips and lapses.

This heterogeneous category of incidents included problems with (1) the user interface (pick list error where an incorrect drug was selected from the drop down menu, errors related to intravenous and enteral feed infusion rates, confusion over the prescription of required therapy in electronic format, and inappropriate use of free-text type-in drug leading to wrong default for route of administration of medicine); (2) treatment duplication, where the same medicine was prescribed both regularly and as 'once only' resulting in two doses being given, or nearly given, close together in time; this was the result of either moving from paper based to electronic prescribing or an over-reliance on the clinical decision support to highlight a potential for treatment duplication; (3) alerts causing a distraction and interruption of workflow, leading to the incorrect medicines being given to a patient; and (4) the log-in/ log-on process which led to a prescribed dose being signed off electronically by a nurse who was still logged onto the system while it had actually been given by another nurse who was not logged on.

• ***Incidents related to training***

Four incidents were training related (5.5%) and involved new users, such as junior doctors being unfamiliar with how to prescribe a particular infusion; agency nurses who were not trained in using PICS and unable to access an appropriately trained staff nurse, leading to the omission of drugs; and staff being unable to carry out administrative tasks on PICS out of hours. One of the incident report narrative described how the reporter felt very "let down" by what he or she felt was a "poor electronic prescribing system" and the consequence was that a patient missed an important drug. This is an example of where a prescriber was unable to use the system to carry out an action that the system was indeed designed to perform. Another training issue was identified in an incident that involved enteral feeding regimes. Nurses on the ward were confused that the electronic system stated 'continuous' indicating no break in feed administration, resulting in the patient being given too much enteral feed. An annotation had been placed on the system, but had not been acted upon. The manner in which these incidents were reported suggests that the reporter was more likely to blame the prescribing system than to identify their own lack of knowledge or lack of practice of using it.

• ***Incidents related to a mixed economy of prescribing systems***

Eight incidents (11%) were identified which related to both electronic and paper-based systems of prescribing being used for a patient being treated in different areas of the hospital, resulting in near misses, the omission of medication, or doses being given too close together. This mixed economy also caused problems in communication between departments and individual clinicians. The critical point at which errors occurred was the handover of patients from non-PICS areas to PICS areas. For example, an allergy to penicillin was documented on paper in the emergency department, but was not transferred to the electronic system when the patient was admitted to the ward. The lack of this information being transcribed across to PICS meant that a penicillin-like antibiotic was prescribed and the patient had an allergic reaction. Paper persistence also played a part in facilitating an error in which a doctor changed a prescription for an antibiotic from an intravenous to an oral route while the nurses were preparing the intravenous medicines, working from a paper print-out of the old prescription. The result was that the patient received an oral as well as an intravenous dose before the error was spotted.

• ***Incidents related to prescribing privileges***

Two incidents (2.7%) involved prescribing privileges on PICS. In one incident, a member of medical staff was unable to prescribe a required medication due to access rights for the system, leading to the patient not receiving treatment. The incident report transcription indicated that the staff member believed that the restrictions imposed by the system were too confining and resulted in "poor patient care". The second incident involved a junior nurse being unable to change the rate of intravenous fluid administration on the system, a task that has to be carried out by a registered, PICS trained nurse, resulting in the actual administration being correct, but not being registered on PICS.

### Effects of time and days of the week on sociotechnical incidents

**Figure 1:**
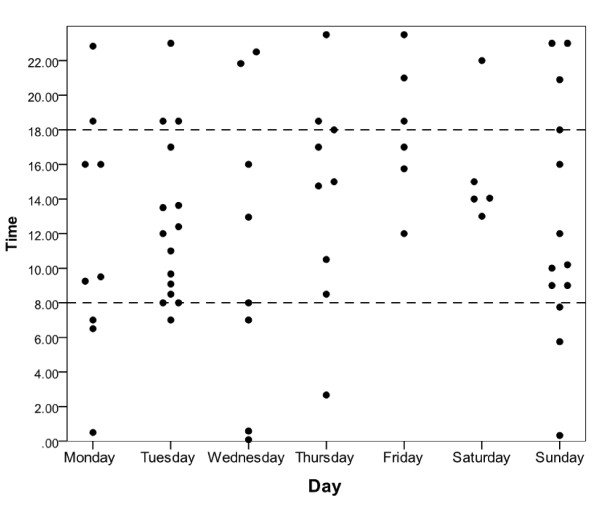
**Scatter plot of the time of sociotechnical incidents occurring during the week**.

Given the scarcity of evidence about medication incidents that may have been an unintended consequence of the introduction of the electronic prescribing system, we further explored the characteristics of these incidents. The proportions of reported incidents which were sociotechnical were compared over different parts of the week and times of the day. The proportion of sociotechnical incidents occurring within office working hours (08:00 - 18:00 inclusive on both weekdays and weekends) were compared to the proportion occurring outside this period. A Fisher's Exact test showed no significant difference (p = 0.892) suggesting that incidents occurring outside office hours were no more likely to be sociotechnical than those occurring at other times. To illustrate this graphically, the sociotechnical incidents were plotted by time and day. This demonstrated no strong clustering of incidents, giving no reason to suspect that any systematic issues were occurring. A number of incidents did not have a recorded time (10%) and were therefore not included in figure 1.

**Figure 2:**
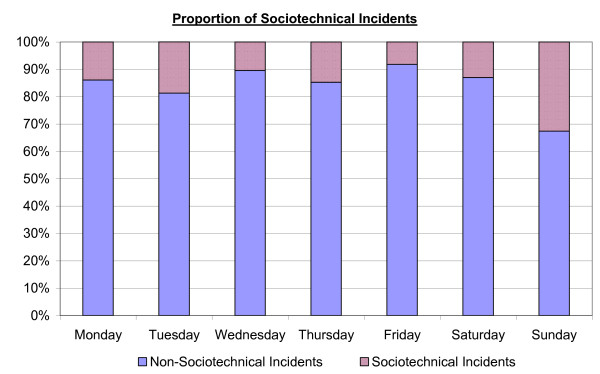
**Breakdown of the sociotechnical incidents occurring during the week**.

As well as illustrating how the frequencies of sociotechnical incidents change over time, figure 2 also shows how the proportion of incidents of this type changed over the days of the week. These data indicate that the number of sociotechnical incidents being reported was not constant over the week. A Chi squared test was conducted in order to test the significance of this observation. The result gave evidence that the proportion of reported incidents which were sociotechnical was not the same on every day (p = 0.013). Figure 2 illustrates the proportions of incidents which were reported on each day of the week that were sociotechnical. This highlights a discrepancy between Sunday and the remainder of the week.

### Multivariable analysis of time and day

Time of day and day of week were then included in a multivariable analysis in order to detect any underlying interaction. Both variables were entered into a backwards logistic regression, including an interaction term. The interaction was found to be insignificant, and so was removed from the initial model. Hence, the test gave no evidence that the likelihood of an incident occurring outside office hours being sociotechnical was different on different days of the week. In the resulting model, whether an incident occurred out of office hours was also found to be insignificant, and so this factor was also removed from the analysis. The final model found that the day of the week was a significant predictor of the likelihood of an incident being sociotechnical (p = 0.02). The Odds Ratios showed no significant difference in the likelihood of an incident being sociotechnical on Tuesday through to Saturday, in comparison with Monday (p-values 0.28-0.78) as shown in table 1. However, this was not the case on Sunday - Odds Ratio 2.8 (95% CI: 1.33-7.67, p = 0.01). Therefore, the conclusion of the multivariable analysis matched that of the univariable analysis, in that the day of the week was the only known factor to have a significant effect on the likelihood of an incident being sociotechnical, with the greatest proportion of such incidents occurring on a Sunday.

**Table 1:** Multivariable Analysis

			95% Confidence Interval for Odds Ratio	
			
	**Sig**.	Odds Ratio	Lower	Upper
Day of the Week	0.020	-	-	-

Tuesday	0.413	1.446	0.598	3.498
Wednesday	0.520	0.720	0.265	1.957
Thursday	0.776	0.868	0.327	2.301
Friday	0.276	0.549	0.187	1.613
Saturday	0.382	0.600	0.191	1.884
Sunday	0.031	2.800	1.101	7.124

Constant	0.000	0.185	-	-

**Figure 3:**
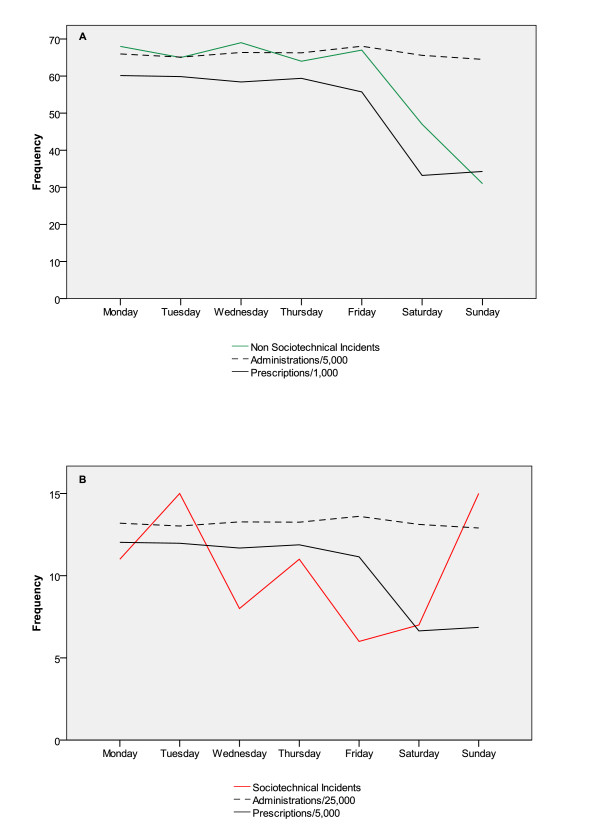
**Frequency of total number of medication administrations and prescriptions for (a) non sociotechnical incidents and (b) sociotechnical incidents**.

In addition, the total number of sociotechnical and non-sociotechnical incidents was plotted against the day of the week on which they occurred. The numbers of medication prescriptions and administrations on each day were also plotted to give an indication of how the level of activity fluctuated over the course of the week. Figure 3 shows that compared to the rest of the week, the level of prescribing activity was considerably lower during the weekend. The frequency of non-sociotechnical incidents (Figure 3a) matched this trend, also falling to a lower level during Saturday and Sunday. In contrast, the frequency of sociotechnical incidents (Figure 3b) increased during the weekend, especially on Sunday. This discrepancy between prescribing activity and the frequency of sociotechnical incidents appears to indicate that the increase in the level of sociotechnical incidents on a Sunday may be of interest as it coincides with a reduction in both non-sociotechnical incidents and prescribing activity.

## Discussion

### Potential for new errors

From descriptive accounts of errors in incident reports we have been able to identify cases where clinical staff interacting with an electronic prescribing system have been involved with errors, i.e. sociotechnical incidents. Out of the total number of incidents reported during the survey period, sociotechnical incidents form a very modest proportion of all medication incidents error (n = 73 15%), but are the third highest type of medication errors reported.

The subcategories of sociotechnical incidents require some added explanation. Half of these related to electronic signatures which may not be that surprising given that overall the PICS system records 120,000 administrations every week in the study hospital. Missing signatures in 36 cases reported from approximately 2.5 million administrations represents a tiny proportion of the total. In the same way that a nurse may be distracted on a paper-based system and forget the last step in the process to sign the chart, this is just as possible in an electronic system although the open patient record on the computer screen should reduce rather than compound this error. If nurses get logged out of the system due to a distraction that takes them away from the computer terminal for a period of time, they will lose any prompt to sign for that medication. It is suspected that lapses within the workflow during the administration process results in discrepancy between the reporter's planned action and the system logs. This type of incident can be regarded as an error substitution, insofar as such an incident could have occurred equally using a paper system when a written signature had not been placed against a prescribed dose. Indeed, environmental factors such as interruptions during the process of medication administration by colleagues, patients or telephone calls have been suspected of contributing to medication administration errors [25]. This has been confirmed by Westbrook et al. [26] who reported that the occurrence and frequency of interruptions were significantly associated with the incidence of procedural failures and clinical errors. However, whereas incidents related to the omission of signatures against the administration of paper-based prescriptions were rarely quantified, PICS renders these incidents visible. This heightened visibility makes the suspected omission of medicines amenable to intervention to help mitigate potential harm to patients associated with missed or multiple doses. It also has the potential to be used as an instrument to monitor staff behaviour.

Administration/ prescribing incidents relate to various different types of incidents, but most could be considered to be technical slips - for example pick list errors, or administering under the wrong log-in. These types of errors are undoubtedly only seen due to the presence of an ICT system, but are not unique to the study hospital, and are well described in the literature as the unintended consequences of ICT [7,9]. Errors that can be attributed to a lack of knowledge about the system formed only a small proportion of the sociotechnical incidents. However, there tends to be an absence of self- awareness around lack of knowledge and competence with regard to prescribing by junior doctors [27].

Paper persistence in institutions with ICT systems has also been implicated in sociotechnical incidents in other reports in the literature [28,29]. The problem of paper persistence was manifest in two forms: first, through a mixed economy of prescribing systems in the same organisation where the roll out of the electronic prescribing system across the hospital has not been completed. Inevitably, when there is the possibility for two parallel systems to interact, there is the potential for errors to arise where these systems contradict, or fail to align. The second problem relates to paper use as a cognitive aid and temporary display device for information, resulting in a lack of alignment between the electronic system and established paper based work practices that staff have adopted locally to organise their work load. In the study hospital, it is made clear to all users that the PICS' prescribing record is the primary record for prescription and medication administration. Errors such as this would not have occurred if the policy of using the electronic prescribing system as the primary of source of medication documentation had been followed. However, this finding might indicate a need for an improvement in the design of the PICS to better support clinical work by eliminating the need for a paper process, or by increasing the number of terminals or portable devices available to staff in clinical areas.

Restricted privileges exist within the system in order to reduce the risk of errors with high-risk medicines, or from untrained staff. Both privilege-related incidents did not result in harm, but in one report it is possible to infer from the description that despite being untrained in intravenous administration (and therefore not having the privileges to document these activities within the PICS system), the nurse in question did alter intravenous medication notwithstanding the restrictions. This was not actually a technical violation as in this case there was no possibility of working around the restrictions within the system. However, clear breaches of systems designed to promote safety need to be examined to ascertain whether underlying them were social, organisational or work flow factors influencing 'work-around' behaviour. Such evidence will inform system design and training. Violations of system restrictions which compromise patient safety will require policies to curb this practice when necessary.

### Timings and frequency of potential new errors

The evaluation of sociotechnical incidents by time of day and day of week suggests a trend for increased proportions of these types of incidents occurring on Sundays, this being almost three times as common as other days of the week. This is an interesting finding and there are several possible explanations. The staff at weekends may not be representative of those usually working in the hospital during the week. It is possible that there is an increased proportion of agency or temporary staff working at weekends who are less familiar with the electronic systems. However, the specificity of Sundays, rather than both days of the weekend, weakens this argument. It is also possible that this effect relates to the presence of fewer technical, pharmacy, senior clinical or managerial staff on duty on Sundays who, at least in many circumstances, could deal with some of the incidents reported before they actually became adverse incidents. There may be other unexplained reasons for the increased proportions of sociotechnical incidents, including changes in the pattern of the non-sociotechnical incidents which affect the denominator, for example, because the pharmacy is closed, thus artificially inflating the proportion. Therefore, this increased proportion may be the result of a combination of different factors including fewer staff on duty and reliance on temporary staff. We would have expected similar results during other out-of-hours times, which was not the case. However, given the under-reporting of medication incidents and the likelihood of inaccuracies in the data provided by reporters about the exact time and date the incident took place, these findings must be treated with caution.

### Limitations

The sample consisted of reported incidents only; the actual (including unreported) number of incidents for this period of time is unknown. It is widely recognised that most medication errors are not reported [30,31]. However, it is reasonable to assume that incidents are reported at a nationally comparable rate at the study hospital [24] and therefore the patterns emerging in this data set are likely to be transferable. We also acknowledge the limitations of conducting quantitative analyses of data extracted from incident reports given the under-reporting of safety incidents. As discussed, although incident reporters are asked to provide the time and date the incident occurred, it is possible that reporters give the time and date the incident was identified or entered onto the incident reporting system, thus inadvertently providing inaccurate data.

This was a single-site, short term study, focusing on an organisation that has developed its own electronic prescribing system, and our results are therefore not directly applicable to other sociotechnical systems. It is important to consider further analysis which could be conducted to examine coalescing factors such as frequency of drug rounds during specific times, and also note that in analysing reported incidents in particular there may be a lag in the time the incident occurred and the time the incident has been reported leading to failures in memory and inaccuracies. In the context of a critical absence of empirically tested models for achieving large scale change to improve patient safety, evidence is needed to increase our understanding of why some organisations successfully implement ICT supported work practices while others fail [32]. Thus more attention needs be given on how these results scale up and this will require multi-site studies. On the other hand, Greenhalgh et al [14] conclude that further detailed study of the often hidden clinical work that is carried out by nurses, junior medical staff and health care assistants - which Ellingsen and Monteiro [33] refer to as 'situated micro-practices' is needed in order to generate knowledge about how ICT can be designed to fit into collaborative clinical practice and team communication. This study of medication incidents related to the sociotechnical context has shed some light on these 'situated micro-practices'.

## Conclusions

This study reports on the analysis of medication incidents at a large acute hospital over a period of five months. We were specifically interested in errors in the processes of prescribing and administering medication to in-patients involving the electronic prescribing system. From descriptive accounts provided by the reporters of incidents, we have been able to identify cases where clinical staff interacting with the system have been involved with errors, i.e. sociotechnical incidents. These form a modest proportion of all medication errors, but they indicate that introducing an electronic prescribing system has the potential to give rise to new types of risks to patient safety. These included pick list juxtaposition errors; the confusion generated by a mixed economy of paper-based and electronic prescription systems; uncertainty as to whether patients have received a dose of their medication as the responsible nurse's electronic signature was not recorded on the system; and distractions and interruptions to workflows caused by features such as the timing out of log-ons. Being aware of these types of errors is important to the clinical and technical implementers of such systems in order to, where possible, design out unintended problems, highlight training requirements, and revise clinical practice protocols.

## Competing interests

The authors declare that they have no competing interests.

## Authors' contributions

This study formed part of a larger evaluation led by JC. SR and JC developed the original idea for this study. SR designed the study, and SR and AR collected the data. Data analysis was conducted by all authors. The manuscript was prepared by SR with additional contributions by all authors. All authors read and approved the final manuscript.

## Pre-publication history

The pre-publication history for this paper can be accessed here:

http://www.biomedcentral.com/1472-6947/11/29/prepub
